# Risk of mature B‐cell neoplasms and precursor conditions after joint replacement: A report from the Haematological Malignancy Research Network

**DOI:** 10.1002/ijc.32765

**Published:** 2019-11-23

**Authors:** Eleanor Kane, Daniel Painter, Alexandra Smith, Maxine Lamb, Steven E. Oliver, Russell Patmore, Eve Roman

**Affiliations:** ^1^ Epidemiology and Cancer Statistics Group, Department of Health Sciences University of York York United Kingdom; ^2^ Hull York Medical School York United Kingdom; ^3^ Queens Centre for Oncology, Castle Hill Hospital Hull United Kingdom

**Keywords:** lymphoma, myeloma, monoclonal gammopathy of uncertain significance, monoclonal B‐cell lymphocytosis, joint replacement

## Abstract

Associations between previous joint replacement and B‐cell lymphoid malignancies have been reported, but despite numerous reports, associations with the disease subtypes have received little attention. Using a UK‐based register of haematological malignancies and a matched general population‐based cohort, joint replacements from linked hospital inpatient records were examined. Cases diagnosed 2009–2015 who were aged 50 years or more were included; 8,013 mature B‐cell neoplasms comprising myeloma (*n* = 1,763), diffuse large B‐cell lymphoma (DLBCL, *n* = 1,676), chronic lymphocytic leukaemia (CLL, *n* = 1,594), marginal zone lymphoma (MZL, *n* = 957), follicular lymphoma (FL, *n* = 725) and classical Hodgkin lymphoma (CHL, *n* = 255), together with monoclonal gammopathy of uncertain significance (MGUS, *n* = 2,138) and monoclonal B‐cell lymphocytosis (MBL, *n* = 632). Odds ratios (OR) and 95% confidence intervals (95%CI) were calculated relative to 10 age‐ and sex‐matched controls using conditional logistic regression. Having had a joint replacement before diagnosis was associated with myeloma (OR = 1.3, 95% CI 1.1–1.5, *p* = 0.008) and MGUS (OR = 1.3, 95% CI 1.1–1.5, *p* < 0.001). Excluding replacements in the year before diagnosis, the MGUS risk remained, elevated where two or more joints were replaced (OR = 1.5, 95% CI 1.2–2.0, *p* = 0.001), with hip (OR = 1.2, 95% CI 1.0–1.5, *p* = 0.06) or knee replacements (OR = 1.5, 95% CI 1.2–1.8, *p* < 0.001). Associations with CHL and two or more replacements (OR = 2.7, 95% CI 1.3–5.6, *p* = 0.005) or hip replacements (OR = 1.9, 95% CI 1.0–3.4, *p* = 0.04); and between DLBCL and knee replacements (OR = 1.3, 95% CI 1.0–1.6, *p* = 0.04) were also observed. Our study reports for the first time a relationship between joint replacements and MGUS; while absolute risks of disease are low and not of major public health concern, these findings warrant further investigation.

## Abstract 2

**What's new?**

While lymphoid malignancies are increased in persons with previous joint replacements, data on associations with particular diagnostic subtypes is lacking. Here, the authors investigated the relationship between joint replacement and mature B‐cell neoplasms and their precursor conditions in an established cohort of patients with hematological malignancies linked to national healthcare records. Previous joint replacement was associated with subsequent elevated risk of myeloma, monoclonal gammopathy of uncertain significance, and Hodgkin lymphoma subtypes of hematological disease. While the findings indicate that absolute risks are low, joint replacement procedures are increasing and disentangling the underlying reasons behind these associations warrants further investigation.

**Abbreviations**
CHL: classical Hodgkin lymphoma
CI: confidence interval
CLL: chronic lymphocytic leukaemia
DLBCL: diffuse large B‐cell lymphoma
FL: follicular lymphoma
HES: Hospital Episodes Statistics
HMDS: Haematological Malignancy Diagnostic Service
HMRN: Haematological Malignancy Research Network
ICD‐O3: International Classification of Diseases for Oncology version 3
IgM: immunoglobulin M
MBL: monoclonal B‐cell lymphocytosis
MGUS: monoclonal gammopathy of undetermined significance
MZL: marginal zone lymphoma
NHS: National Health Service
OR: odds ratio
WHO: World Health Organization

## Introduction

Including chronic lymphocytic leukaemia (CLL), myeloma and more than 90% of lymphomas, mature B‐cell malignancies account for around 60% of all haematological cancers.^1^, ^2^ With diverse epidemiological features, treatment pathways, and outcomes these cancers comprise a heterogeneous group of over 50 subtypes.^1^ For some subtypes, environmental risk factors are well established; including biological (e.g. certain infections), physical (e.g. ionising radiation) and chemical (e.g. pesticides) agents.^3^, ^4^, ^5^, ^6^, ^7^ A number of familial predisposition syndromes and genetic risk factors have also been implicated, as have several acquired comorbidities (e.g. autoimmunity) and drugs/procedures associated with immunosuppression (e.g. organ transplantation).^8^, ^9^, ^10^, ^11^, ^12^, ^13^ Currently, however, known risk factors only account for a relatively small proportion of the total mature B‐cell cancer burden.

With a view to gaining additional insight into the pathogenesis of mature B‐cell malignancies, epidemiological research is increasingly focusing on the molecular aspects of these complex malignancies, as well as exposures and conditions that interact with the immune system.^14^, ^15^, ^16^, ^17^, ^18^, ^19^ In this regard, precursor lymphoproliferative conditions, which have been shown to share some of the same risk factors as their malignant counterparts, are also of interest; the two most notable being monoclonal gammopathy of undetermined significance (MGUS) and monoclonal B‐cell lymphocytosis (MBL).^20^, ^21^, ^22^ Both of these asymptomatic clonal disorders are associated with subsequent progression to mature B‐cell malignancies: non‐IgM and light‐chain MGUS to myeloma (at a rate of around 1% per year) and the less common IgM MGUS to other lymphoproliferative neoplasms (at rate of about 1.5% per year)^1^, ^21^, ^23^; the high‐count MBL CLL‐type, which accounts for around 75% of the total, progressing to CLL at a rate of around 1–2% a year.^1^, ^22^, ^24^

The present report concerns the potential impact that previous joint replacements may have on the subsequent development of mature B‐cell malignancies and their precursor conditions. Many materials used in orthopaedic implants are suspected carcinogens,^25^ and replacements can produce debris and also inflammation.^26^, ^27^ Accordingly, with the aim of investigating the potential adverse health effects of such procedures, several cohorts of joint replacement recipients have been established; and although findings for all cancers combined have tended to be equivocal, those for mature B‐cell malignancies are more consistent, albeit often based on comparatively small numbers of events.^28^, ^29^, ^30^, ^31^, ^32^, ^33^, ^34^, ^35^, ^36^, ^37^, ^38^ Using a more statistically powerful design, this article presents findings from a case–control study embedded within a population‐based cohort of patients with haematological malignancies (cases) and a matched cohort of unaffected individuals from the general population (controls).

## Methods

Cases were from the UK Haematological Malignancy Research Network (HMRN, www.hmrn.org), a specialist register initiated in September 2004 which provides real‐world data on all haematological cancers and precursor conditions that can be generalised to the UK as a whole.^39^ HMRN collects all diagnoses, including progressions and transformations, reported and coded to the latest WHO ICD‐O3^1^ by clinical specialists.^40^ Set in a catchment population of 4 million people served by 14 hospitals, the network registers ~2,400 haematological malignancy diagnosed each year. In order to facilitate comparisons with the general population, HMRN also has a general‐population cohort; patients diagnosed between January 1, 2009 and December 31, 2015 were each matched on age and sex to 10 unaffected individuals from the same catchment population.^11^ HMRN operates under a legal basis that permits full treatment and outcome data to be collected from clinical records without explicit consent, and all cases and controls are linked to nationwide information on deaths, cancer registrations and Hospital Episode Statistics (HES). This report includes 10,783 patients aged 50 years or over newly diagnosed with a mature B‐cell malignancy or precursor condition between 1 January 2009 and 31 December 2015 and their age‐ and sex‐matched controls (*n* = 107,830). The malignancies included were myeloma (*n* = 1,763), DLBCL (*n* = 1,676), CLL (*n* = 1,594), MZL (*n* = 957), FL (*n* = 725) and CHL (*n* = 255); and the precursor conditions were MGUS (*n* = 2,138) and MBL (*n* = 632); and all controls were assigned a “pseudodiagnosis date” equivalent to the date of diagnosis of their matched case.

Joint replacements are among the most common surgical operations conducted in patients over 40 years.^41^ In England, around two‐thirds of joint replacements are funded by the NHS,^42^ which are recorded with the date of the operation in HES Admitted Patient Care (HES‐APC), regardless of whether they are performed by the NHS or the independent sector. For the present analysis, all joint replacement operations performed between April 1, 1997 and before the date of diagnosis (patient cohort members), or the corresponding pseudodiagnosis date (comparator cohort members) were extracted using OPCS4 codes (OPCS Classification of Interventions and Procedures Version 4 codes^43^) relating to joint replacement operations (Table 1). Focus was on the commonest prosthetics (hip, knee, shoulder, elbow or ankle); with data on primary replacements, resurfacing procedures, revisions, conversions and any other related operation included. Laterality of each procedure was also extracted, with coincident bilateral operations being counted twice, once for each side. As well as examining joint replacements up to diagnosis/pseudodiagnosis, analyses were repeated excluding any joint replacements that occurred in the year or 5 years before diagnosis/pseudodiagnosis. To quantify associations between joint replacements and malignancy, odds ratios (ORs) and 95% confidence intervals (CIs) were estimated using conditional logistic regression. Since cohort members could have replacements in different types of joints, risk estimates for specific joints were adjusted for whether or not they had had replacements in other joints. All analyses were conducted using Stata 15.1.^44^

**Table 1:** OPCS4 Codes for operations involving a joint replacement in the hip, knee, shoulder, elbow or ankle

	OPCS4 Codes	
Joint	Joint‐specific replacement operations	Replacement operations where joint identified separately (+ Z codes)
		(W05, W43–W45, W55, W58, W91.3, W91.8)
Hip	W37–W39, W46–W48, W93–W95	+ (Z75.6, Z76.1–Z76.2, Z76.8–Z76.9, Z84.3, Z84.9)
Knee	W40–W42, O18	+ (Z76.5, Z77.3–Z77.5, Z77.8–Z77.9, Z78.7, Z84.4–Z84.6)
Shoulder	W49–W51, W96–W98, O06–O08	+ (Z69.1, Z81.2–Z81.4)
Elbow	W52–W54, O21–O26	+ (Z70.1, Z81.5)
Ankle	O32	+ (Z85.6, Z85.8)

Operations were recorded using OPCS4 versions 4.2–4.7. Codes for primary joint replacements end with 0.1, 0.8 or 0.9 except in the shoulder, where the codes W50.4, W51.5, W96.5 and W98.6 also define primary replacements/resurfacing. Laterality was identified using the codes Z94.1, Z94.2, Z94.3 for bilateral; right‐ and left‐sided operations, respectively.

## Ethics approval

HMRN has ethical approval from Leeds (West) Research Ethics Committee (reference 04/Q1205/69) and the Health Research Authority Confidentiality Advisory Group under Section 251 of the NHS Act (2006; reference PIAG 1‐05 (h)/2007).

## Results

Joint replacements occurred in 995/10783 (9.2%) cases and 9179/107830 (8.5%) controls prior to the date of diagnosis/pseudodiagnosis. Osteoarthritis was the most frequent indication for the first replacement (75.5% among cases, 76.7% among controls), with fractures accounting for a further 11.3% in both cases and controls. Although no association with joint replacement was observed for all mature B‐cell neoplasms combined (OR = 1.0, 95%CI 1.0–1.1, *p* = 0.33), the risk varied by subtype, being highest for myeloma (OR = 1.3, 95%CI 1.1–1.5, *p* = 0.008) and CHL (OR = 1.4, 95%CI 0.9–2.3, *p* = 0.13), but below or close to unity for DLBCL, MZL, FL and CLL (Table 2). An increased risk was also seen for MGUS (OR = 1.3, 95%CI 1.1–1.5, *p* < 0.001), but not for MBL. The associations with MGUS and CHL remained when operations performed in the 1 or 5 years before diagnosis were removed, but did not for myeloma (Table 2).

**Table 2:** Risk of mature B‐cell neoplasms and precursor conditions after a joint replacement in the hip, knee, ankle, shoulder or elbow up to diagnosis/pseudodiagnosis date or up to 1 or 5 years before diagnosis/pseudodiagnosis date

		Up to diagnosis			Up to 1 year before diagnosis			Up to 5 years before diagnosis		
	Cases *n*	% Cases	% Controls	OR (95% CI)	% Cases	% Controls	OR (95% CI)	% Cases	% Controls	OR (95% CI)
										
*Mature B‐cell neoplasms*										
Total	8,013	8.7	8.4	1.0 (1.0–1.1)	7.7	7.5	1.0 (0.9–1.1)	4.8	4.5	1.1 (1.0–1.2)
Myeloma	1,763	10.2	8.3	1.3 (1.1–1.5)	8.6	7.5	1.2 (1.0–1.4)	5.0	4.5	1.1 (0.9–1.4)
Diffuse large B‐cell lymphoma	1,676	9.4	9.1	1.0 (0.9–1.2)	8.5	8.0	1.1 (0.9–1.3)	5.4	4.7	1.2 (0.9–1.5)
Chronic lymphocytic leukaemia	1,594	7.0	8.1	0.8 (0.7–1.0)	6.3	7.3	0.9 (0.7–1.1)	4.1	4.4	0.9 (0.7–1.2)
Marginal zone lymphoma	957	9.5	8.6	1.1 (0.9–1.4)	8.5	7.7	1.1 (0.9–1.4)	4.8	4.8	1.0 (0.7–1.4)
Follicular lymphoma	725	7.2	7.6	0.9 (0.7–1.3)	6.3	6.7	0.9 (0.7–1.3)	3.2	3.9	0.8 (0.5–1.2)
Classical Hodgkin lymphoma	255	9.0	6.5	1.4 (0.9–2.3)	7.8	5.9	1.4 (0.8–2.3)	6.7	3.5	2.0 (1.2–3.5)
*Precursor conditions*										
Monoclonal gammopathy of undetermined significance	2,138	11.4	9.1	1.3 (1.1–1.5)	10.2	8.1	1.3 (1.1–1.5)	7.0	4.9	1.5 (1.2–1.8)
Monoclonal B‐cell lymphocytosis	632	8.4	8.1	1.0 (0.8–1.4)	7.9	7.3	1.1 (0.8–1.5)	5.1	4.3	1.2 (0.8–1.7)

Odds ratios (OR) and 95% confidence intervals (95% CI) comparing HMRN cases aged 50 or over and diagnosed 2009–2015 to 10 individually age‐sex matched controls were estimated using conditional logistic regression.

**Figure 1:**
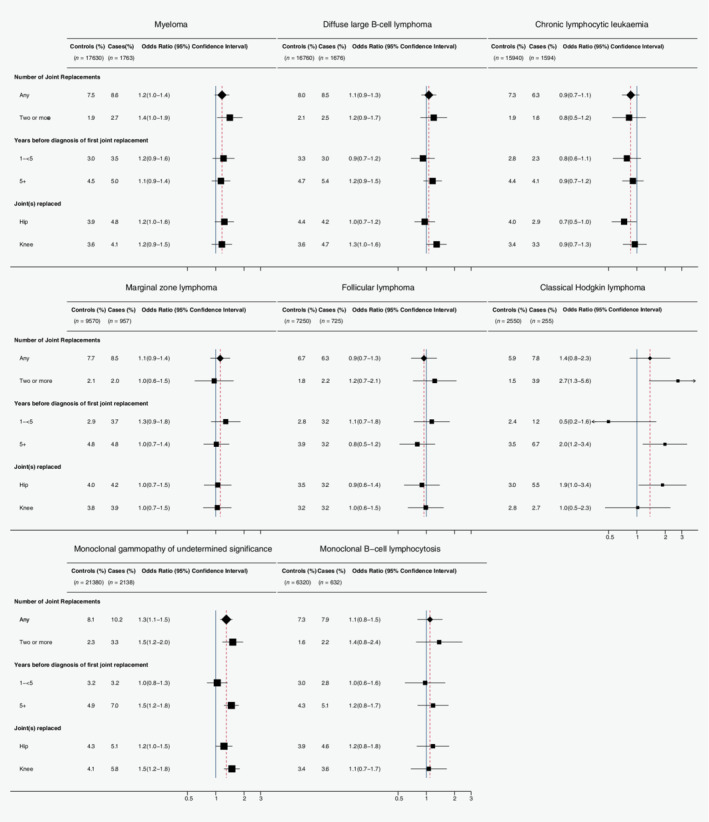
Mature B‐cell neoplasms and precursor conditions diagnosed 2009–2015 aged 50 or over and their age‐sex matched controls with one or more operations involving a joint prosthesis in the hip, knee, ankle, shoulder or elbow up to 1 year before diagnosis/pseudodiagnosis, odds ratios (OR) and 95% confidence intervals (CI), distributed by number of joints replaced, number of years between first joint replacement and diagnosis/pseudodiagnosis and specific joint(s) replaced. Boxes are weighted to the total number of cases and controls. [Color figure can be viewed at wileyonlinelibrary.com]

In the time window up to a year before diagnosis, 8.2% of cases and 7.6% of controls had at least one joint replaced, and among those with replacements, over a quarter had two or more separate joints replaced (29.2% of cases and 26.6% of controls with a replacement). As shown in Figure 1, risks were raised when two or more joints were replaced for myeloma (OR = 1.4, 95% CI 1.0–1.9, *p* = 0.03), MGUS (OR = 1.5, 95% CI 1.2–2.0, *p* = 0.001) and CHL (OR = 2.7, 95% CI 1.3–5.6, *p* = 0.005). For MGUS and CHL, the effect was also found 5 or more years before diagnosis (MGUS: OR = 1.5, 95% CI 1.2–1.8, *p* < 0.001; CHL: OR = 2.0, 95% CI 1.2–3.4, *p* = 0.01). The pattern for myeloma was less clear, as risk estimates were similar for 1 to <5 years and 5 or more years before diagnosis (OR = 1.2, 95% CI 0.9–1.6, *p* = 0.17; OR = 1.1, 95% CI 0.9–1.4, *p* = 0.27, respectively).

As expected most operations were on hip or knee joints, and, in almost equal numbers; less than 5% were performed on the shoulder, elbow or ankle joints. MGUS and myeloma risks were raised after both hip and knee replacements (MGUS: OR = 1.2, 95% CI 1.0–1.5, *p* = 0.06 and OR = 1.5, 95% CI 1.2–1.8, *p* < 0.001; myeloma: OR = 1.2, 95% CI 1.0–1.6, *p* = 0.08 and OR = 1.2, 95% CI 0.9–1.5, *p* = 0.24 for hip and knee, respectively). For CHL, the risk was increased with hip (OR = 1.9, 95% CI 1.0–3.4, *p* = 0.04) rather than knee replacements (OR = 1.0, 95% CI 0.5–2.3, *p* = 0.96; Fig. 1). Among other subtypes, associations were generally lacking, exceptions being an increased risk of DLBCL after knee replacement (OR = 1.3, 95%CI 1.0–1.6, *p* = 0.04) and of MZL with shoulder replacement (OR = 2.6, 95%CI 1.2–5.6, *p* = 0.02). Although those who had a joint replacement tended to be older at diagnosis/pseudodiagnosis than those who had not (mean age 77.2, 95% CI 76.7–77.8 compared to 71.6, 95% CI 71.4–71.8), risks did not vary by age, or by sex either (data not shown).

Findings for MGUS among those with IgM (*n* = 342) and non‐IgM (*n* = 1,655) subtypes are shown in Table 3. For IgM MGUS, the risk estimate with any joint replacement was slightly higher (OR = 1.7, 95% CI 1.2–2.4), but not significantly different from that for non‐IgM MGUS (OR = 1.3, 95% CI 1.1–1.5). Similarly, we found little difference between the two MGUS subtypes when the number of primary joint replacements, the time before the diagnosis of the first replacement, and whether hip or knee joints were replaced were examined.

**Table 3:** Risk of monoclonal gammopathy of uncertain significance by subtype after a joint replacement in the hip, knee, ankle, shoulder or elbow up to 1 year before diagnosis/pseudodiagnosis date

	MGUS			IgM MGUS			Non‐IgM MGUS		
	Controls (%)	Cases (%)		Controls (%)	Cases (%)		Controls (%)	Cases (%)	
	*n* = 21,380 (100)	*n* = 2,138 (100)	OR (95% CI)	*n* = 3,420 (100)	*n* = 342 (100)	OR (95% CI)	*n* = 16,550 (100)	*n* = 1,655 (100)	OR (95% CI)
*Number of joint replacements*									
None	19,647 (91.9)	1,920 (89.8)	1 (ref)	3,161 (92.4)	301 (88.0)	1 (ref)	15,202 (91.9)	1,485 (89.7)	1 (ref)
Any	1,733 (8.1)	218 (10.2)	1.3 (1.1–1.5)	259 (7.6)	41 (12.0)	1.7 (1.2–2.4)	1,348 (8.1)	170 (10.3)	1.3 (1.1–1.5)
Two or more	482 (2.3)	71 (3.3)	1.5 (1.2–2.0)	76 (2.2)	15 (4.4)	2.1 (1.2–3.8)	380 (2.3)	55 (3.3)	1.5 (1.1–2.0)
*Years before diagnosis of first joint replacement*									
1 to <5	685 (3.2)	69 (3.2)	1.0 (0.8–1.3)	114 (3.3)	15 (4.4)	1.4 (0.8–2.4)	523 (3.2)	52 (3.1)	1.0 (0.8–1.4)
5+	1,048 (4.9)	149 (7.0)	1.5 (1.2–1.8)	145 (4.2)	26 (7.6)	1.9 (1.2–3.0)	825 (5.0)	118 (7.1)	1.5 (1.2–1.8)
*Joint(s) replaced*									
Hip	911 (4.3)	108 (5.1)	1.2 (1.0–1.5)	133 (3.9)	20 (5.8)	1.6 (1.0–2.6)	721 (4.4)	84 (5.1)	1.2 (1.0–1.5)
Knee	866 (4.1)	125 (5.8)	1.5 (1.2–1.8)	134 (3.9)	25 (7.3)	2.0 (1.3–3.1)	668 (4.0)	96 (5.8)	1.5 (1.2–1.8)

Odds ratios (OR) and 95% confidence intervals (95%CI) comparing HMRN cases aged 50 or over and diagnosed 2009–2015 to 10 individually age‐sex matched controls were estimated using conditional logistic regression.

## Discussion

This is the first study to describe associations between previous joint replacements across the spectrum of mature B‐cell malignancies and precursor conditions. Using a population‐based case–control design, this large record linkage study with over 8,000 cases with mature B‐cell neoplasms, 2,700 with precursor conditions and 10 times as many controls found associations between joint replacements and subsequent MGUS, myeloma and CHL. We found that the risk of both MGUS and CHL were increased with joint replacements several years before diagnosis, while that for myeloma was present with replacements in the year before diagnosis and less so with replacements at earlier times; this latter observation leaving open the possibility that some procedures may have been carried out on patients whose myeloma had not yet been detected. In all cases, the associations increased with increasing numbers of replacements; MGUS and myeloma were associated with both hip and knee, whereas CHL was primarily linked with hip. Neither DLBCL nor MZL were associated with joint replacements overall, although increased risks were observed for knee and shoulder replacements, respectively. Consistent with the heterogeneity of B‐cell neoplasms, we found no associations for the other most common subtypes.

In contrast to most population‐based registers, HMRN has world‐class centralised diagnostics, following strict condition‐specific criteria for accuracy and consistency across the spectrum of haematological malignancies; MGUS, for example, is diagnosed with the presence of neoplastic plasma cells in the bone marrow in addition to detectable paraprotein in peripheral blood. Our study was also specifically designed to make robust comparisons between patients with haematological malignancies and the general population; HMRN's controls comprise age‐ and sex‐matched individuals randomly sampled from the study's catchment population, and both HMRN's cases and controls are linked to the same nationwide administrative databases. This case–control approach, novel among the studies of joint replacement recipients,^28^, ^29^, ^30^, ^31^, ^32^, ^33^, ^34^, ^35^, ^36^, ^37^, ^38^ yielded large numbers of cases in well‐defined diagnostic categories. With healthcare data available from April 1, 1997, our study captured primary replacements for any major joint through to the date of diagnosis/pseudodiagnosis (January 1, 2009, to December 31, 2015), and so unlike others, was able to examine associations with the number, as well as specific joints replaced, as well as the time since the first replacement.

Prior studies have followed joint replacement recipients from their first hip or knee replacement for average periods of around 4–8 years postoperation.^28^, ^29^, ^30^, ^31^, ^32^, ^33^, ^34^, ^35^, ^36^, ^37^, ^38^ In agreement with national data on joint replacements,^45^ we found that the median age at the first joint replacement was around 70 years of age; no differences of note were detected between cases and controls, or by disease subtype. Seventy years of age is also around the median age at which B‐cell neoplasms are diagnosed, apart from CHL where the median age at diagnosis is 41.^2^ We did not find associations in the year leading up to diagnosis, with the exception of myeloma; increased risks were instead found where the first joint was replaced five or more years before the diagnosis. Consequently, joint replacement recipients tended to be diagnosed with a B‐cell condition at later ages (mean age of 77.2); with our analysis restricted to persons aged 50 or over, the comparison for CHL was a mean age of 76.5 among recipients compared to 67.2 overall. In the absence of associations closer to diagnosis (1 to <5 years), these observations are consistent with a longer latency between joint replacement and the diagnosis of a B‐cell condition.

While our study has many strengths, weaknesses may have arisen through the use of HES data to define our exposure. In England, hospital data are available back to the year 1997 and for our cohorts, this timeframe covered more than a decade of secondary healthcare records before diagnosis/pseudodiagnosis (2009–2015). We believe this is sufficient coverage to identify the majority of individuals aged 50 or over who had previous major prosthetic work recorded in HES. HES records operations funded by the NHS in England, so joint replacement operations performed outside England or funded privately cannot be accounted for. Although the impact of the former may be minimal, privately funded replacements comprise a reasonable proportion of all major joint replacement operations, currently around a third of the total.^42^ However, since case and control distributions of socioeconomic status at the time of the first joint replacement were similar, and in contrast to some other cancers, haematological malignancies are not related to socioeconomic status,^2^ cases having joint replacements conducted outside the NHS more (or less) often than controls seems unlikely. A criticism common to previous studies is the healthy recipient effect, whereby persons undergoing major surgery to replace a joint are healthier than the general population, introducing bias in prospective cohorts at the point of ascertainment. Here, individuals were ascertained at the time of haematological malignancy diagnosis/pseudodiagnosis typically several years postimplant, minimising any healthy recipient effect except perhaps in the year prior, where no decreased risks were found. Our reliance on OPCS clinical codes for joint replacements meant that the type of prosthetic could not be specified; metal‐on‐metal prosthetics (MoM) are one type to have received attention, but where MoM recipients were compared to those who received other types, the risk of lymphoproliferative cancer was found to be no different.^46^, ^47^, ^48^, ^49^

In this first study to examine whether joint replacements are related to mature B‐cell neoplasms and precursor conditions, we found the most consistent associations for myeloma, MGUS and CHL. Across these three subtypes, associations were, on the whole, present after joint replacements several years before diagnosis; with multiple primary replacements; and with replacements in the hip and knee joints. Contrary to these observations, we found little or no evidence that joint replacements were associated with the other subtypes, namely DLBCL, MZL, CLL, FL and MBL. Conducted in a period when the number of joint replacements performed is increasing,^50^ our findings must be considered in the context of low absolute risk of any of these diagnosis and should not raise major public health concerns; nevertheless, whether the prosthetics or the underlying reasons for the procedures are the likely explanation warrants further investigation.

## Data Availability

The data that support the findings of our study are available from the corresponding author upon reasonable request.
